# When less is more: The way forward for mental health interventions during the perinatal period

**DOI:** 10.1371/journal.pmed.1004138

**Published:** 2022-12-13

**Authors:** Mark Tomlinson, Mary Jane Rotheram-Borus

**Affiliations:** 1 Institute for Life Course Health Research, Department of Global Health, Stellenbosch University, Cape Town, South Africa; 2 School of Nursing and Midwifery, Queens University, Belfast, United Kingdom; 3 Semel Institute Department of Psychiatry and Biobehavioral Sciences, University of California at Los Angeles, Los Angeles, California, United States of America

Globally, almost 40 million people are living with HIV, with almost half of these being women of childbearing age [1]. In addition, depression is a leading cause of disability globally, but despite these high levels, data for low- and middle-income countries (LMICs) is limited—particularly in the context of HIV [2]. Given this context, the work of Kaaya and colleagues [3] describing a peer-facilitated psychological group intervention (Healthy Option) for perinatal women living with HIV and receiving ART is welcome. The study was a cluster randomised controlled trial conducted in urban districts of Dar es Salaam. Utilising community-based health workers trained in problem solving and cognitive behaviour therapy for women screened for depression risk. While the Healthy Options intervention showed no benefit for a reduction in depressive symptoms at 9 months postpartum (primary outcome), there was a benefit for depressive symptoms at 6 weeks postpartum [3].

This is an important study adding to the growing evidence base of interventions for improving mental health in the perinatal period in LMICs. Having said this, there are a number of key areas that we believe bear discussion in thinking about the way forward for the design and evaluation of perinatal mental health interventions such as this one. In this commentary, we focus on 4 aspects: (1) screening for as opposed to diagnosing depression; (2) continued creation of new evidence-based interventions (EBI) versus building on existing evidence of common elements/principles, approaches; (3) applying EBI universally versus stepped care approaches; and (4) integrated versus siloed approaches.

## Screening vs. diagnosis

The extent to which screening actually improves detection or management of depression remains contested [4]. A considerable concern, particularly in LMICs, is using screening tools to determine provision of referral and/or treatment. It has been estimated that while screening tools may miss less than 3% of people with a potential diagnosis of depression, fewer than 50% of those screening positive, in fact, have depression [5]. Using a screening tool in routine primary health care settings in resource-constrained health systems may overwhelm fragile health systems and direct already limited resources away from people with an actual diagnosis [6]. Screening in LMIC countries must ensure high specificity, as the costs of diverting large numbers of “false positive” women into care is problematic [6,7]. Kaaya and colleagues [3] expected that the lower rates of post-birth depression reflected remission of depression. Yet, there are few longitudinal studies from LMICs showing rates of depression remission postnatally. In our own work [8], we found comparable rates of antenatal and postnatal depression within the first 6-month post-birth; postnatal rates were higher, in fact, than antenatal depression. A systematic review of African women, both those living with HIV and not, found about a 10% lower rate of postnatal depression, compared to prenatally [9]. These are substantially lower remission rates than those observed in the Kaaya and colleagues study [3].

## Novel EBI vs. common elements approaches

Over 10 years ago, Chorpita and colleagues identified the shared practice elements of EBI for child and family mental health treatments [10]. Identifying the common, shared approaches adopted across multiple EBI is increasing. By 2019, the US Preventive Services Task Force validated the utility of perinatal EBI for depression [11], a finding which was confirmed with a meta-analyses of 50 EBI in 2021 [12]. More than 80% of EBIs have been found to share 14 common practice elements—highlighting the potential of training community health workers and/or mental health counsellors how to use these 14 elements, rather than focus on replicating a sequenced set of activities and scripts and developing new interventions.

## Necessary and sufficient EBI—Stepped-care approaches

Stepped-care approaches are key to avoiding intensive costs. Initial screening can be followed by a diagnostic interview to determine diagnosis and/or the severity of the depression in order to inform treatment [13,14]. The finding in the Kaaya and colleagues study that the benefits of the intervention at first follow up were attenuated in the context of interpersonal violence and HIV-related stigma, adds weight to the need for a more nuanced approach to screening and treatment.

## Integrated vs. siloed approaches

The argument of Kaaya and colleagues [3] about how women living with HIV require specialised mental health services, punctuates this need. Given the stigma and added burden of living with a chronic infectious disease, providing a tailored intervention to all women living with HIV may appear at first blush to be a good approach. However, most perinatal depression interventions adopt cognitive-behavioural theoretical (CBT) approaches [9,11] and HIV-related interventions, including those identified by the US Centers for Disease Control and Prevention (https://www.cdc.gov/hiv/effective-interventions/index.html) also share roots in CBT. The shared theories, change strategies (i.e., practice elements), and principles [10] of both perinatal depression and HIV-related interventions, as well as the stigmatising features of both depression and HIV suggest that more generic, trans-condition strategies are required. What is not needed is a plethora of additional siloed mental health services tailor made based on comorbidities such as HIV, long COVID, or cardiovascular disease. While the Healthy Options model includes a number of common elements, it is still branded as a “new intervention.” We would argue that in a global health landscape of diminishing resources, the field does not require novel interventions. Instead, using common elements, principles, or transdiagnostic approaches should be the norm. The novel data needed will come from researching optimal implementation strategies and how to shape these interventions to local contexts.

Kaaya and colleagues have provided an EBI that appears efficacious in the short term. The current scientific norms require that Healthy Options would need to replicated at least twice and then tested in a large effectiveness trial in order to be a genuine EBI [15]. The intervention would then join the ranks of potential EBI for perinatal depression. Instead of celebrating the short-term efficacy of another CBT EBI, perhaps this study leads us to recognise that, similar to other perinatal depression interventions, health systems must implement strategies to screen, diagnose, and treat perinatally depressed mothers with sequentially more intensive CBT interventions. Healthy Options is one nugget supporting this approach.
